# Ideal Spintronics in Molecule-Based Novel Organometallic Nanowires

**DOI:** 10.1038/srep12772

**Published:** 2015-08-04

**Authors:** Qilong Sun, Ying Dai, Yandong Ma, Wei Wei, Lin Yu, Baibiao Huang

**Affiliations:** 1 School of Physics, State Key Laboratory of Crystal Materials, Shandong University, Jinan 250100, People’s Republic of China; 2 Engineering and Science, Jacobs University Bremen, Campus Ring 1, 28759 Bremen, Germany

## Abstract

With the purpose of searching for new intriguing nanomaterial for spintronics, a series of novel metalloporphyrin nanowires (M-PPNW, M = Cr, Mn, Fe, Co, Ni, Cu and Zn) and hybrid nanowires fabricated by metalloporphyrin and metal-phthalocyanine (M-PCNW) are systematically investigated by means of first-principles calculations. Our results indicate that the transition metal atoms (TMs) embedded in the frameworks distribute regularly and separately, without any trend to form clusters, thus leading to the ideally ordered spin distribution. Except for the cases embedded with Ni and Zn, the others are spin-polarized. Remarkably, the Mn-PPNW, Mn-PCNW, MnCu-PPNW, MnCr-PCNW, and MnCu-PCNW frameworks all favor the long-ranged ferromagnetic spin ordering and display half-metallic nature, which are of greatest interest and importance for electronics and spintronics. The predicted Curie temperature for the Mn-PCNW is about 150 K. In addition, it is found that the discrepancy in magnetic coupling for these materials is related to the competition mechanisms of through-bond and through-space exchange interactions. In the present work, we propose not only two novel sets of 1D frameworks with appealing magnetic properties, but also a new strategy in obtaining the half-metallic materials by the combination of different neighboring TMs.

Recently, molecular spintronics with improved performance and enhanced functionality have attracted increasing attention as they show great promise for the next generation of electronic devices^1^,^2^,^3^,^4^,^5^,^6^,^7^,^8^. Among them, organic spintronics is an important subject, in which the spin-polarized signal can be mediated and manipulated by employing various organic molecules^6^. When comparing with the conventional metals or semiconductors, the unquestionable advantages of the weak spin-orbit and hyperfine interactions in organic molecules would lead to considerably longer spin relaxation length and spin-lifetime, which are crucial for the performance of the spintronic devices. In addition, there are many other unique advantages of the organic materials, such as cheap, low-weight, mechanically flexible as well as chemically interactive^5^,^9^. With these remarkable merits, organic spintronics is regard as one of the most promising alternatives to conventional silicon-based electronics^10^. Nevertheless, not all organic molecules could meet the requirements of the electronic devices. It is always an essential issue to design and synthesize those “nontrivial” organic molecules, which can be assembled into spintronics for specific functions.

On the other hand, with the trend toward miniaturization in spintronics, low dimensional materials have been considered to be the best solutions since graphene was first fabricated in 2004^11^,^12^. After that, a great deal of effort has been made to search for other potential nanomaterials, which can be used in the spintronic devices. For practical applications, an ideal nanomaterial should have ordered spin signals; in this sense, the materials with regularly and separately distributed transition metal atoms (TMs) would be highly desirable. Moreover, it has been demonstrated that the intriguing half-metallicity is also favored in the spin filters^13^,^14^. Unfortunately, only few such organic/inorganic nanomaterials have been reported either theoretically or experimentally. Thus, novel frameworks, which could be fabricated by a simple synthetic method, are urgently required. So far, several one-dimensional (1D) molecular wires including 1D TM–cyclopentadienyl^7^, TM–benzene^15^, TM–anthracene^16^, and TM–phthalocyanine^10^ wires have been proposed. In particular, porphyrin and phthalocyanine derivatives, which share the similar molecular configurations, are well-known as ideal candidates for molecular electronic devices. For example, the 1D Zn-porphyrin arrays have already been achieved experimentally and exhibit promising applications as a conducting molecules^17^. Some interesting questions then arise: can the 1D nanowires formed by the connected metalloporphyrin and metal-phthalocyanine possess the long-ranged spin coupling and intrinsic half-metallic properties at the same time? Is there any possibility to introduce interesting magnetic properties between different neighboring TMs in the designed configurations? With these questions, we attempt to design novel 1D nanomaterials with practical application for spintronic devices by employing organometallic molecules. Thus, not only for the theoretical design, but also for the further applications, this work is highly desirable.

In the present study, the electronic and magnetic properties of two sets of 1D nanowires (denoted as M-NW, M = Cr, Mn, Fe, Co, Ni, Cu and Zn), see Fig. 1, as well as the magnetic properties with various combination of neighboring TMs, are investigated by means of first-principles calculations. It is shown that the TMs dock regularly and separately in all the proposed frameworks, and the magnetic moments are 4.0, 3.0, 2.0, 1.0 and 1.0 *μ*_B_ for Cr, Mn, Fe, Co, and Cu, respectively. Furthermore, our calculations reveal that the proposed Mn-PPNW, Mn-PCNW, MnCu-PPNW, MnCr-PCNW, and MnCu-PCNW frameworks show the nature of long-ranged ferromagnetic spin ordering and display half-metallic feature at the same time, suggesting that these frameworks are promising material platforms for future applications in spintronics. Taking Mn-PCNW as an example, the Curie temperature (T_C_) is estimated by means of Monte Carlo (MC) simulations, and the possibility of long-ranged cooperative magnetic order near 150 K is found. Further, it is noticed that the mechanism of the magnetism can be well understood by our previously proposed competition mechanisms between through-bond and through-space exchange interactions. Motivated by these results, we propose two kinds of highly desired 1D frameworks, which could be used in future spintronics applications and offer a new efficient avenue for controllable and tunable spintronic devices by adopting different neighboring TMs. Based on our theoretical results and the previous prepared nanowires^17^,^18^,^19^,^20^,^21^,^22^, we expect that the intriguing magnetic properties of the mentioned 1D frameworks revealed in this work will arise further experimental efforts in achieving enhanced spintronics.

## Results and Discussion

**Figure 1:**
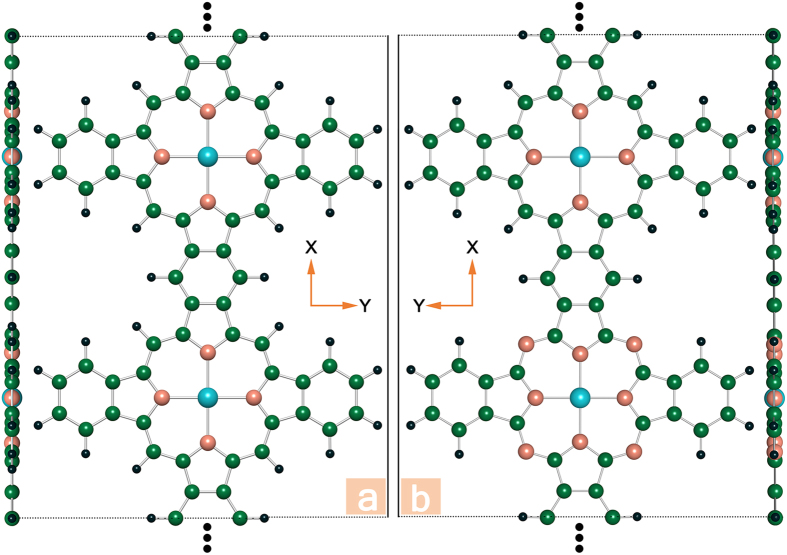
Side and top views of the two considered geometric configurations: (**a**) M-PPNWs and (**b**) M-PCNWs, which is the hybrid metalloporphyrin and metal phthalocyanine nanowires. The periodic direction of the 1D nanowires is along the X-axis. The H, C, N and TM atoms are highlighted in black, green, orange and cyan, respectively.

The geometric structures of 1D M-PPNW and M-PCNW frameworks (M = Cr, Mn, Fe, Co, Ni, Cu and Zn) are shown in Fig. 1. The corresponding structural parameters are summarized in Table 1. As shown in Fig. 1, these 1D molecular nanowires are straight without any distortions, strongly implying that the studied frameworks are good building blocks for fabricating 1D molecular chains. On the other hand, the side views shown in Fig. 1 indicate an ideal planar nature for the M-PPNW and M-PCNW frameworks, which is similar to the pristine graphene. These particular geometric structures make it possible to realize uniform frameworks. More interestingly, when the molecules are assembled into the M-PPNWs and M-PCNWs, the 1D molecular wires with regularly separated TMs are formed without any tendency to form clusters, see Fig. 1. Those are favorable in achieving the long-standing dream of the ideal nanostructures for spintronic devices. To further confirm the final geometries, we also examine the configurations with clustered TMs in the nanowires. Our results reveal that the clustering of TMs will significantly increase the energy of the system, indicating that the configurations with separated TM atoms are relatively more stable. Furthermore, the molecular dynamics (MD) simulations are carried out to check the stability of these designed nanowires. The results indicate that the structures of the studied nanowires are stable even at the temperature of 300 K. The stability of the structures enables these materials to be applied at or even above room temperature, which is critical for potential electronic devices. In addition, some geometric distortions at different levels are observed after the MD simulations, while the TMs also maintain the regular distributions in the frameworks. However, in view of the previous experimental results, the nanowires would be achieved on a supporting substrate, thus the distortions could be avoided.

As listed in Table 1, the metal-nitrogen distance varies slightly from Cr to Zn for both M-PPNW and M-PCNW frameworks. For the case of M-PPNWs, the two values of the D_0_ correspond to the bond lengths which are defined as the distance between the central metal atom and two unequal nitrogen atoms along Y-axis and X-axis, respectively. Firstly, it can be seen that the values decrease monotonically with the atomic number increasing from Cr (2.07/2.03 Å) to Ni (1.98/1.98 Å), and then increase until Zn (2.11/2.03 Å). This result is in good agreement with the experimental data and theoretical investigations for the corresponding molecules^23^. There are several factors which are responsible for this trend, i.e., the variations of the atomic radii and the electronegativity. Besides, except for those two bonding electrons, the number of remaining valence electrons in the TMs, which fill the bonding orbitals, increases from four to eight for Co to Ni, causing the increasing stability. For Cu and Zn, the number of remaining valence electrons surpasses eight and, subsequently, the additional electrons will fill the anti-bonding orbital, leading to the elongated bond length. Secondly, it is interesting to note that, for each M-PPNW (M = Cr, Mn, Fe, Co, Ni, Cu and Zn), the metal-nitrogen distance along the X-axis is slightly smaller than the distance along the Y-axis, which is different from our previous studies on the 2D metalloporphyrin monolayer. It is worth emphasizing that the minor Jahn−Teller distortions occur because of the different coordination spheres between the X-axis and Y-axis, leading to remove the degeneracy and lower the energy. For M-PCNWs, the similar trends could also be identified. The values of D_1_ and D_2_ correspond to the band lengths of metalloporphyrin and metal-phthalocyanine in the same cell, respectively. We also note that the metal-nitrogen distance in metal-phthalocyanine ring is smaller than the values in the metalloporphyrin ring in the same M-PCNW, which means the metalloporphyrin is in an expanded geometry compared with the metal phthalocyanine. In detail, the optimized bond lengths selected for all of the structures are listed in Table 1.

Besides the favorable geometric properties, interesting magnetic properties are supposed to be observed in such two intriguing configurations in comparison with TM-doped conventional dilute magnetic semiconductors, where the doped TM atoms tend to form clusters^24^,^25^, thus leading to nonintrinsic magnetism and low Curie temperature values. So it is extremely challenging to prevent the TMs from clustering because of the strong d-d interactions. Since the frameworks are the derivatives of isolated porphyrin and phthalocyanine molecules, we first focus on the magnetic properties of these isolated molecules. According to our calculations, we find that, except for Ni and Zn, which are spin-unpolarized, the other TMs will lead to a spin-polarized ground state when assembled into metalloporphyrin and metal-phthalocyanine. And for these two types of transition metal complexes, the magnetic moments of Cr, Mn, Fe, Co, Ni, Cu, and Zn are approximately 4, 3, 2, 1, 0, 1, and 0 *μ*_B_, respectively. The spin configuration of a specific transition metal complex molecule is completely determined by the occupation of the 3d orbitals of central metal atom, while the valence electron configuration for TM = Mn-Ni (and Zn) are 3d^n^4s^2^ with n = 5–8 (and n = 10); for Cr and Cu, the configuration are 3d^5^4s^1^ and 3d^10^4s^1^, respectively. These results are in good agreement with our previous studies^10^,^26^.

Now a question arises as to whether the magnetic properties of these two transition metal complex molecules can be affected when they are connected into 1D wire, since the isolated molecules are magnetic. To answer this question, we systematically discuss the electronic and magnetic properties of 1D M-PPNW and M-PCNW molecular wires based on their ground-state structures. The main results concerning the electronic and magnetic properties of the 1D M-PPNW and M-PCNW frameworks are summarized in Table 1, including the magnetic moments, the energy difference (E_ex_) between ferromagnetic (FM) and antiferromagnetic (AFM) states, and the energy gap between the conduction band minimum (CBM) and the valence band maximum (VBM). Interestingly, the calculated magnetic moment per unit cell of these two considered 1D configurations is still about 4, 3, 2, 1, 0, 1, and 0 *μ*_B_ for Cr-NW, Mn-NW, Fe-NW, Co-NW, Ni-NW, Cu-NW and Zn-NW respectively, which is similar to their corresponding isolated molecules discussed above. This result indicates that the influence of junctions on the magnetic moment can be ignored in the 1D M-PPNW and M-PCNW frameworks, which should be a consequence of the long-distance-induced weak interaction between the TMs. And the magnetic moment is mainly contributed by the central TM atom, while the other atoms contribute only a little to the magnetic moment, which will be discussed in detail in the following. Furthermore, based on the results above, we can conclude that the magnetic properties can be controlled by the inclusion of different combinations of the TMs. Therefore, this tunable magnetic behavior may offer more potential in the applications in future magnetic storage materials and spintronics.

However, the existence of magnetic moment does not necessarily result in FM spin ordering. It is thus essential to study the magnetic coupling between the neighboring TMs. Then the supercells containing two repeating units are adopted for both 1D M-PPNW and M-PCNW frameworks. Depending on the initial spin configurations, we consider the following three magnetic coupling configurations: (a) ferromagnetic (FM) coupling; (b) antiferromagnetic (AFM) coupling; (c) nonmagnetic (NM) state. Based on this model, the magnetic interactions of these differently coupled systems could be deduced by the exchange energy (*E*_*ex *_*= E*_*FM*_*–E*_*AFM*_). The values of *E*_*ex*_ per unit are listed in Table 1. Positive (negative) exchange energy indicates that the ground state of the system is AFM (FM). Results of our calculations show that Mn-PPNW, Mn-PCNW, Fe-PPNW, and Co-PCNW favor FM couplings, with the energy of 16, 68, 85, and 183 meV per unit lower than that of the corresponding AFM states, respectively. This result indicates that, although the coupling distances between the TMs are separated by about 10 Å, these systems still could display interesting ferromagnetic properties. Different from M-PCNW, the case of M-PPNW is more complex. In detail, the Co-PPNW favors the nature of paramagnetic with the *E*_*ex *_= 0 meV, while the nanowires embedded with Ni and Zn are nonmagnetic (Table 1); on the other hand, for Cr-PPNW, Cr-PCNW, Fe-PCNW, Cu-PPNW and Cu-PCNW, the ground state is FM. As seen from the computational results, the spin ordering is not only affected by the introduced TMs, but also greatly influenced by the configuration of the 1D system. Interestingly, it turns out that our new designed frameworks assembled with different organic molecules is very desirable in searching for the 1D nanomaterials possessing the ferromagnetic coupling. The different coupling manners between these cases might originate from the mechanism of the magnetism. It should be noted that the long-range magnetic interactions found in these 1D wires are very interesting and important, endowing the M-PPNW and M-PCNW systems with great potential in spintronics.

**Figure 2:**
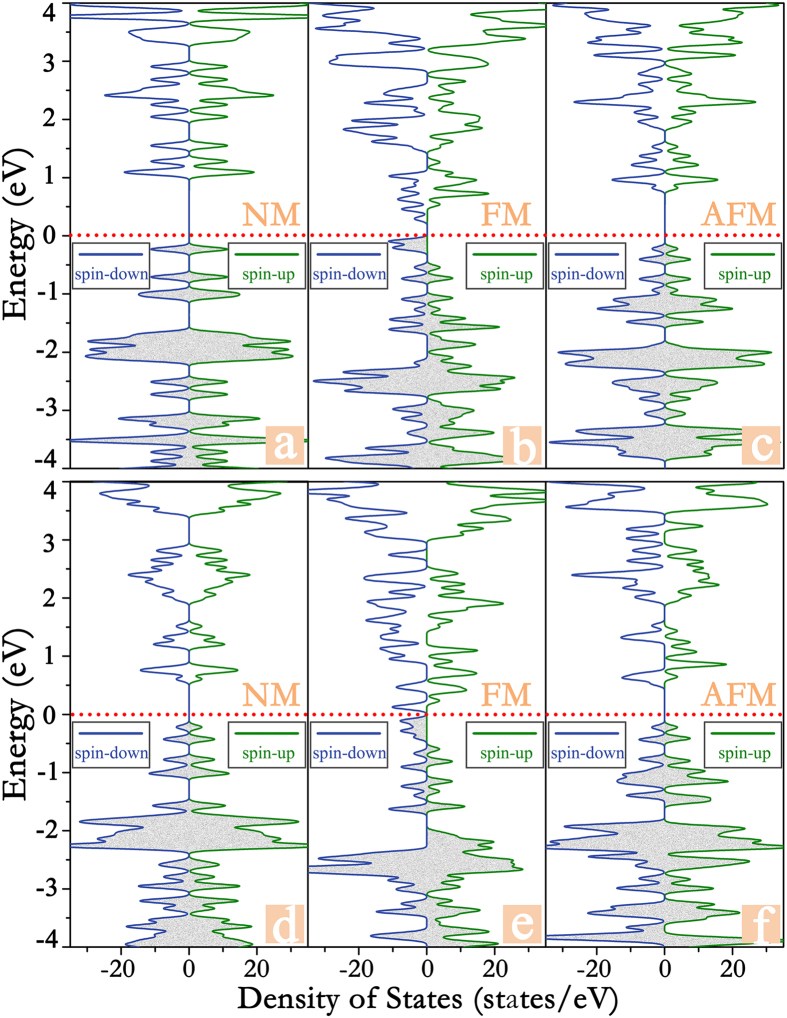
Total density of states (DOS) of (**a**) Zn-PPNW, (**b**) Mn-PPNW, (**c**) Cr-PPNW, (**d**) Zn-PCNW, (**e**) Mn-PCNW, (**f**) Cr-PCNW. The horizontal dashed line represents the Fermi level.

**Figure 3:**
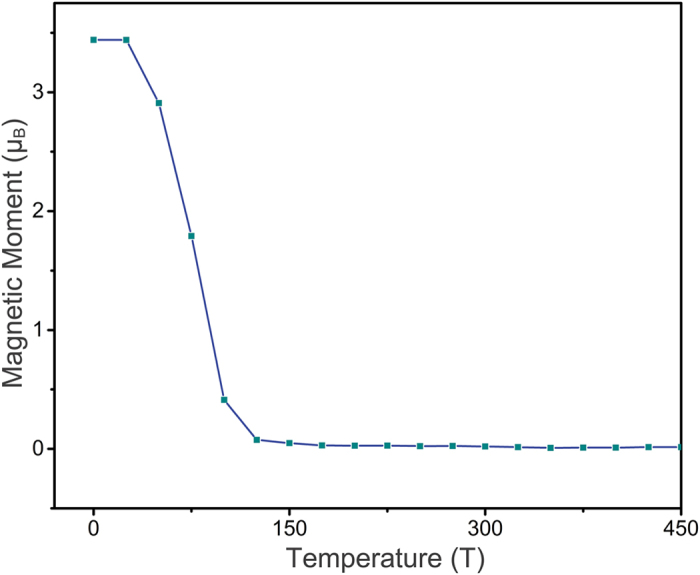
The variation of the total spin magnetic moment per unit cell of 1D Mn-PCNW framework with the temperature. The transition from ferromagnetic to paramagnetic state occurs at 150 K.

Taking Cr, Mn, and Zn with their corresponding PPNW and PCNW configurations as examples, we plot the spin-resolved total density of states (TDOS) in Fig. 2, and the occupied density of states are highlighted by the fill color of gray. Depending on the spin states with lowest energy, the TODS of the frameworks embedded with Cr, Mn and Zn correspond to the ground states of antiferromagnetism, ferromagnetism and nonmagnetic, respectively. As illustrated in Fig. 2a,d, the majority and minority spin channels of the 1D Zn-PPNW and Zn-PCNW frameworks display similar distributions, implying that these two Zn centered frameworks have no spin polarization, which is consistent with the results above. While for 1D Cr-PPNW framework, the symmetric total spin states are attributed to the AFM magnetic coupling. In contrast, the unoccupied TDOS of the Cr-PCNW framework are asymmetric while the magnetic moment of the whole system is 0 *μ*_B_. On the other hand, it is clear that, the semiconducting nature could be found for the 1D Zn-PPNW, Zn-PCNW, Cr-PPNW, and Cr-PCNW with calculated energy gaps of 0.96, 0.49, 0.71, and 0.42 eV (summarized in Table 1), respectively. So, after the introduction of metal-phthalocyanine in the 1D PPNM, the calculated energy gaps of the 1D nanowires have been reduced significantly. It is important to note here that those 1D nanowires assembled by different molecules have excellent properties with regularly separated 3d TMs as well as the uniform nanowires. Furthermore, it is exciting that, for the Mn-PPNW and Mn-PCNW frameworks, the majority channel possesses a large band gap, whereas the minority one does not show an obvious gap. Thus the charge transport is dominated by the minority electrons, and the current flow in such a system should be fully spin-polarized. In other words, most significantly, the highly desirable half-metallicity is obtained in the 1D Mn-PPNW and Mn-PCNW frameworks. It is necessary to stress that the half-metallicity, which possess metallic and semiconducting behaviors for different spin channels, have attracted considerable research interests as a key ingredient in high performance spintronics applications, especially as a source of spin-polarized carriers. Though a certain number of half-metallicity have been proposed^27^, it is still important to search for more materials with the promising half-metallicity, especially in low dimensional materials. Therefore, these findings highlight two new promising half-metallic materials toward realistic spintronic applications. Next, taking the Mn-PCNW as an example, we further use the Ising model along with the Monte Carlo (MC) simulation to get an estimation of the Curie temperature (T_C_), which is a phase transition critical point of the system between ferromagnetic and paramagnetic states. T_C_ is an important parameter to evaluate magnetic properties and applicable conditions of the considered systems. The Hamiltonian could be written as


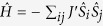


where the 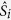 and 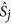 are the magnetic moments at sites *i* and *j*, respectively. 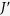 is defined as the exchange parameter, which is determined form the exchange energy E_ex_ using the equation 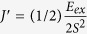, where 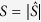 and the factor of 1/2 is adopted as the sum of the magnetic coupling interactions is two in one supercell. In the simulation, a 100 × 100 supercell has been adopted, and the spins on all sites in the supercell changed according to the spin states of the Mn. The T_C_ value appears at the temperature of 150 K for the Mn-PPCW framework, where the magnetic moment per unit cell has been dropped dramatically from 3 *μ*_B_, as vividly shown in Fig. 3. This value of the calculated T_C_ is as high as the reported 2D materials^28^.

**Figure 4:**
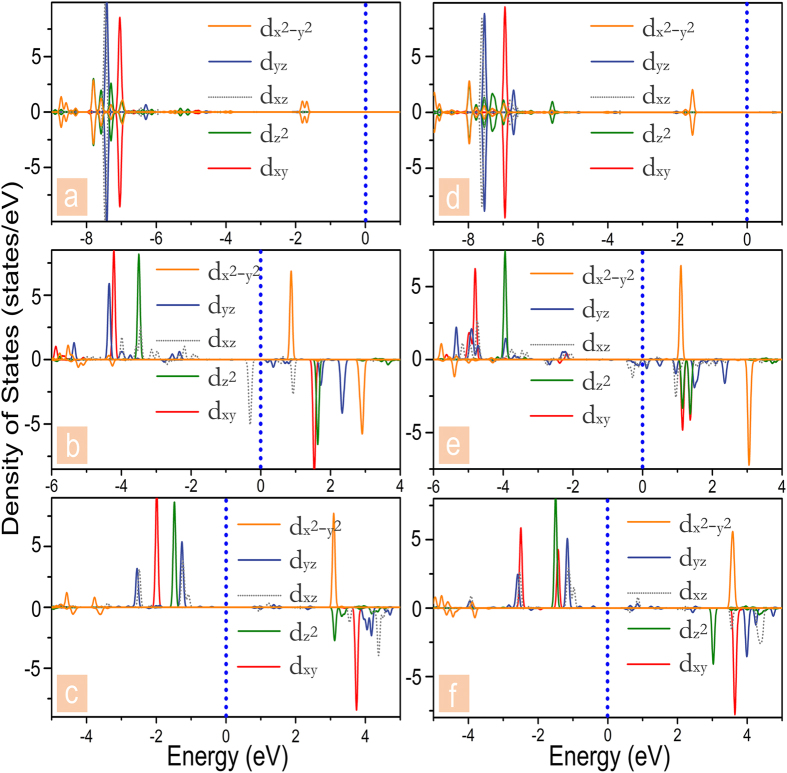
Calculated projected DOS of the d orbitals on the TM atom in 1D (**a**) Zn-PPNWW, (**b**) Mn-PPNW, (**c**) Cr-PPNW, (**d**) Zn-PCNW, (**e**) Mn-PCNW, and (**f**) Cr-PCNW framework. A positive (negative) value corresponds to majority (minority) spin state. The vertical dashed lines (blue) represent the Fermi level.

As mentioned above, the magnetic moments for the considered frameworks mainly come from the unpaired valence electrons of the central TMs. Then, let us still take the above six mentioned frameworks, namely Zn-PPNW, Zn-PCNW, Mn-PPNW, Mn-PCNW, Cr-PPNW, and Cr-PCNW, as examples to reveal the magnetic properties in detail, and the projected density of states (PDOS) of the d electrons onto the TMs of the selected frameworks in the ground state is presented in Fig. 4. For the selected frameworks, the central TMs donate two electrons, forming TM^2+^, to the surrounding molecular rings (referred as to MR^2−^). Under these preconditions, the charge distribution of the two 1D framework can be represented as [∞(MR^2−^) (TM^2+^) (MR^2−^)∞]. Due to the formation of the alternating electronic configuration, the electrostatic interactions within the 1D [∞(MR^2−^) (TM^2+^) (MR^2−^)∞] are strongly strengthened and it became more stable. Similar conclusion has been made in our previous work^26^. Next, in the square-planar-coordinated crystalline field environment, the anisotropic 3d-orbitals usually split into d_xy_, d_z^2^_, d_x^2^-y^2^_, and doubly degenerate d_xz_, d_yz_ orbitals, which is also named “4 + 1” splitting, and the minor spin states can be occupied only after filling four major spin states^21^. As shown in Fig. 4a,d, for Zn-PPNW and Zn-PCNW, the majority and minority states show exactly the same distribution, and all the PDOSs peaks are under the Fermi level, which means the d orbitals are fully occupied by ten d-electrons (Zn^+2^: 3d^10^), leading to nonmagnetic nature. In the cases of Cr-PPNW and Cr-PCNW, the majority spin states of d_xy_, d_z^2^_ d_xz_, and d_yz_ are located below the Fermi level, while the other six states are occupied by holes, rendering these two Cr centered frameworks possess the magnetic moment of 4 *μ*_B_. When the centered TM^2+^ is replaced by the Mn^2+^, which owns the valence configuration of 3d^5^, the one additional spin-down electron would fill orbital of the minority d_xz_ (as presented in Fig. 4b,e). Hence, with respect to the Cr^2+^ systems, the magnetic moment of the corresponding Mn^2+^ systems decreases to 3 *μ*_B_. More remarkably, the metallic feature of the Mn-PPNW and Mn-PCNW in the spin down channel near the Fermi level arises mainly from contribution of d_xz_ and d_yz_, which have delocalized π character. As mentioned above, all the studied Mn^2+^ systems have the nature of half-metallicity, so the delocalized states near the Fermi level ensure the further practical applications.

At this point, it can be known that, for the novel M-PPNW and M-PCNW frameworks considered here, only neighboring Mn atoms could make the systems to favor long-ranged ferromagnetic spin ordering and display half-metallicity, simultaneously. Hence, the 1D Mn-PPNW and Mn-PCNW frameworks, when carrying spin-polarized currents, would work as highly effective spin filters. It is worthy to note that most of the related studies, including our work above, are limited to the configurations with introducing only one type of the neighboring TMs. Now, another idea arises as to whether the different types of neighboring TMs in the considered frameworks can achieve the ferromagnetism and half-metallicity. Since the Mn atom is identified to be a viable candidate for achieving excellent materials used in spintronics in many other researches^10^,^26^,^28^,^29^, subsequently, we explore the configurations of M_1_ (Mn) and M_2_ (Cr, Fe, Co, and Cu) in the same 1D nanowires, while the corresponding frameworks are referred as M_1_M_2_-PPNW and M_1_M_2_-PCNW. Utilizing the previously well-tested *U* (4 eV) and *J* (1 eV), the structures of M_1_M_2_-PPNW and M_1_M_2_-PCNW (Fig. 1) are fully optimized anew. Here, for the M1M2-PCNW nanowires, we also take into account the two different embedded schemes, where the M_1_ and M_2_ atoms could be exchanged between the porphyrin ring and phthalocyanine ring. It is found that MnCr-PCNW and MnCu-PCNW nanowires are more stable in the configurations with Mn embedded in the phthalocyanine ring, while the MnFe-PCNW and MnCo-PCNW nanowires present the opposite cases. The configurations of M_1_M_2_-PCNW with lower energy are adopted in the following calculations. Similar to the studied frameworks above, structural relaxation suggests that these two 1D frameworks with different neighboring TMs are also planar without any buckling. And the calculated electronic and magnetic properties are summarized in Table 2. The magnetic moments of Mn, Cr, Fe, Co, and Cu are approximately 3, 4, 2, 1, and 1 *μ*_B_, respectively, which is consistent with the frameworks with same type TM. Furthermore, interesting magnetic properties are also observed in 1D M_1_M_2_-PPNW and M_1_M_2_-PCNW. Apart from the corresponding collocations of MnFe and MnCo, which are antiferromagnetic (AFM), the other two kinds of frameworks energetically favor ferromagnetic (FM) states. Especially for the 1D MnCu-PPNW framework, the energy difference between FM and AFM states is about 85 meV, indicating that the MnCu-PPNW would be robustly ferromagnetic at room temperature. This tunable magnetic behavior may offer more space in applications for future magnetic storage materials and spintronics.

**Figure 5:**
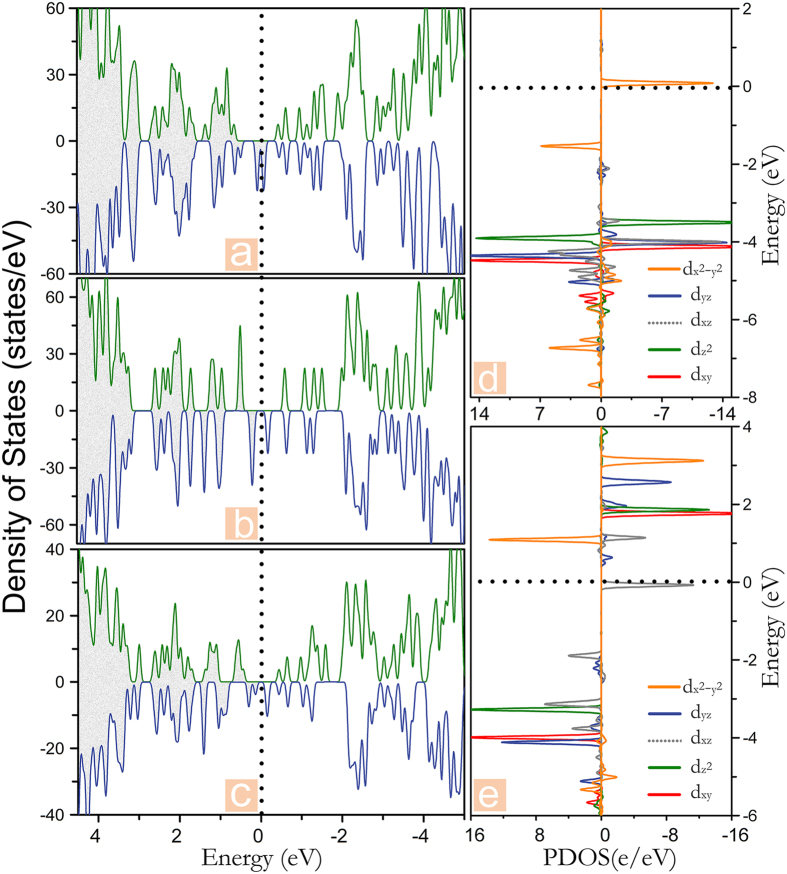
Total density of states (DOS) of (**a**) MnCu-PPNWW, (**b**) MnCu-PCNW, (**c**) CrMn-PCNW frameworks. The (**d**) and (**e**) represent the energy splitting of the 3d orbitals of Cu and Mn, respectively, by projected DOS in the selected MnCu-PPNW framework. A positive (negative) value corresponds to majority (minority) spin state. The black dashed lines represent the Fermi level.

In order to understand the electronic and magnetic properties of the frameworks with different neighboring TMs in detail, we also present the spin-polarized TDOS and PDOS of the three selected frameworks with the ground states of FM, i.e., MnCu-PPNW, MnCr-PCNW, and MnCu-PCNW. As shown in Fig. 5a–c, it is clear that, the spin up channels of the three selected frameworks have a band gap while the spin down channels have partially filled states, indicating that the half-metallicity is realized. And the MnCr-PPNW, MnFe-PPNW, and MnCo-PPNW are semiconductors with the band gap of 0.28, 0.21, and 0.19 eV, respectively, while the MnFe-PCNW and MnCo-PPNW share the metallic properties. Thus, it could be concluded that these three frameworks, i.e., MnCu-PPNW, MnCr-PCNW, and MnCu-PCNW, are also viable choices for designing spin filter. Further, to understand the magnitude of the magnetic moments listed in Table 2, taking the Mn-PCNW as an example, the PDOS of the Cu and Mn have been presented in Fig. 5d,e. In the case of Cu atom, except for the orbital minority d_x^2^-y^2^_, all the spin states are located below the Fermi level, rendering the molecular ring possesses a magnetic moment of 1 *μ*_B_. In contrast, for the neighboring Mn atom, the majority of the spin states of d_xy_, d_yz_, d_z^2^_, and d_xz_ are filled. Meanwhile, the fifth valence election occupies the minority d_xz_, leading to a magnetic moment of 3 *μ*_B_, which is exactly similar to the electron configuration of the framework with same neighboring TMs mentioned above. It verifies that the magnetic properties of the transition metal molecules will not be influential when they are connected into 1D wire. Furthermore, our novel proposed strategy of the combination with different neighbor TMs is feasible.

**Figure 6:**
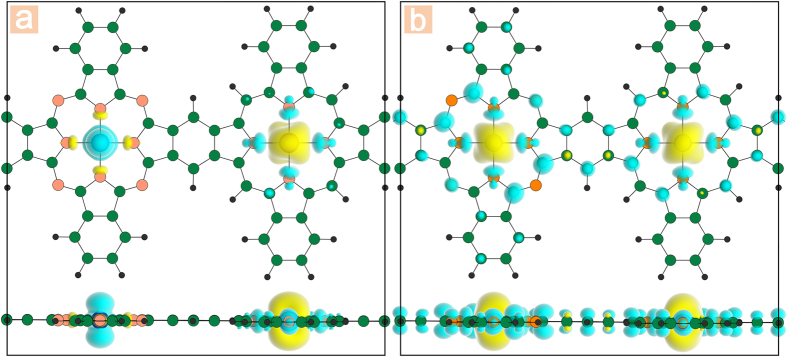
The spin density (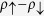) for (**a**) MnCo-PCNW, (**b**) Mn-PCNW with an isosurface value of 0.003  e/Å^3^. Yellow (blue) indicates the positive (negative) values.

At last, we examine the mechanism of the magnetic coupling in these 1D nanowires. Based on the inferences above, there are two electrons of the centered TM that transfer to the molecular ring, forming the [∞(MR^2−^) (TM^2+^) (MR^2−^)∞] alternative configuration, which leads to strong coupling between the TM^2+^ and the MR^2−^. Here, we take the MnCo-PCNW (showing AFM in ground states) and Mn-PCNW (being FM) for example, and the calculated spin-resolved charge density is presented in Fig. 6. We can see that, for both frameworks, the charge carriers localized around the two molecular rings between the TMs are polarized with a small magnetic moment and have the opposite spin orientation with respect to that of the TM atoms. Some similar results have already been reported. Usually, the mechanism of the magnetism originates from the through-bond or through-space exchange interaction. For the MnCo-PCNW (as shown in Fig. 6a), it is defined that the TM atom with up-spin induces down-spin density on its neighbor TM atom directly, no matter a same type TM atom or a different type one, forming a configuration like […↓↑…]. Then the through-space spin polarization become the dominant impact, as the links between the connected metalloporphyrin and metal-phthalocyanine are unspin-polarized. Consequently, the MnCo-PCNW shows antiferromagnetism. As expected from the mechanism of the through-space exchange interaction, when we only consider the isolated TM atom chain by removing the molecular rings, the AFM phase is more stable than the FM phase. On the other hand, for the Mn-PCNW framework, the through-bond spin polarization takes place along direction of the 1D chain, showing a coupling configuration like […↑_↓_↑_↓_…]. Here, the downward arrows represent the two molecular rings between the TM atoms. As shown in Fig. 6b, most of the C toms between the TMs are spin-polarized, and the system favors ferromagnetism. According to this mechanism, the intrinsic difference of the long-ranged magnetic coupling in these nanowires could be easily understood. This mechanism also has implications in the deep understanding of the magnetism of many other organometallic nanowires.

## Conclusion

Two sets of novel 1D nanowires are constructed and their electronic and magnetic properties are studied systematically by means of first-principles calculations. In contrast to the current focused organic metallic low dimensional materials used in spintronic devices, we provide a blueprint for the design of a stable variety of molecular wires. We not only successfully designed two sets of novel 1D frameworks, but also provided a new strategy in obtaining the half-metallic materials by combination of different neighboring TM atoms. It is found that the TMs in the proposed frameworks dock regularly and separately, forming the ordered spin arrangement in the studied systems. Expect for the frameworks embedded with Ni and Zn, the other studied 1D frameworks are magnetic, carrying magnetic moments of 4.0, 3.0, 2.0, 1.0 and 1.0 *μ*_B_ for Cr-NW, Mn-NW, Fe-NW, Co-NW and Cu-NW, respectively. In this sense, the magnetic properties could be easily modified by employing different combinations of TMs. Most importantly, it is noted there are appreciable long-range magnetic interactions in the considered frameworks. Our calculations reveal that Mn-PPNW, Mn-PCNW, MnCu-PPNW, MnCr-PCNW, and MnCu-PCNW frameworks favor long-ranged ferromagnetic spin ordering and display half-metallic, while such long-range magnetic interactions are very interesting and important. Further, we also predict the Curie temperature of the selected Mn-PCNW by applying the Monte Carlo simulations, which is about 150 K. Meanwhile, the MnCu-PPNW has even larger exchange energy, indicating more robust ferromagnetism at room temperature. Additionally, the magnetism in the designed frameworks can be understood by the competition mechanisms of through-bond and through-space exchange interactions. Thus in our examples, for Mn-PCNW, the through-bond is the major mechanism, forming FM coupling. In contrast, the AFM coupling in MnCo-PCNW is attributed to the through-space mechanism. Our results revive these novel systems as viable candidates to overcome all the aforementioned challenges. We hope that the present study will stimulate further experimental effort in this field.

## Methods

The first-principles calculations are performed by density functional theory (DFT) in conjunction with projector augmented wave (PAW) potentials, which is implemented in the Vienna ab initio simulation package (VASP)^30^,^31^. For the exchange-correlation functional, the generalized gradient approximations (GGA) of Perdew–Burke–Ernzerhof (PBE) is used^32^. Due to the well-known problems of standard DFT in describing strongly correlated systems, we use the GGA+U method for the treatment of the partly occupied 3d orbitals of TM atoms^33^. In our GGA+U calculations, correlation energy (U) of 4.0 eV and exchange energy (J) of 1.0 eV for TM d orbitals are adopted, which has been proven well to reproduce the electronic structures and magnesium of organometallic complexes^10^,^26^,^34^. The periodic M-Pp frameworks are optimized within one repeating unit. We applied periodic boundary conditions and a vacuum space of 15 Å along the z direction in order to avoid the interactions between two slabs in the nearest-neighbor unit cells. A cutoff energy of 450 eV is used for the plane wave expansion of the wave function to converge the relevant quantities. Test calculations with larger energy cutoffs ensure that the results are fully converged. The Monkhorst-Pack scheme of k point sampling is used for integration over the first Brillouin zone. A 5 × 5 × 1 grid for k-point sampling is used for geometry optimization and the static total energy calculations^35^. All structures are fully optimized until the residual forces are less than 0.02 eV/Å. The convergence criteria for energy of 10^−5 ^eV is met^36^. In addition, the molecular dynamics (MD) simulations are carried out to examine thermal stability of the nanowires at 300 K within a 3 fs time step^37^,^38^. The 2 × 1 × 1 supercell models consisting of 196 and 188 atoms for M-PPNW and M-PCNW, respectively, are used for the simulations.

## Additional Information

**How to cite this article**: Sun, Q. *et al.* Ideal Spintronics in Molecule-Based Novel Organometallic Nanowires. *Sci. Rep.* **5**, 12772; doi: 10.1038/srep12772 (2015).

## Figures and Tables

**Table 1:** Geometric, electronic and magnetic properties of the 1D M-PPNW and M-PCNW Frameworks.^a^

	**TM**	**Cr**	**Mn**	**Fe**	**Co**	**Ni**	**Cu**	**Zn**
PPNW	D_0_	2.07/2.03	2.03/2.01	2.01/2.00	1.99/1.99	1.98/1.98	2.05/2.02	2.11/2.03
	M_0_	4	3	2	1	0	1	0
	E_ex_	11	−16	−85	0	…	2	…
	E_g_	0.71	half-metal	0.66	0.77	0.69	0.31	0.96
PCNW	D_1_	2.07/2.03	2.03/2.00	2.01/2.00	2.00/1.99	1.98/1.98	2.05/2.02	2.11/2.03
	D_2_	1.99/1.96	1.95/1.93	1.93/1.92	1.92/1.91	1.91/1.91	1.98/1.94	2.03/1.96
	M_0_	4	3	2	1	0	1	0
	E_ex_	11	−68	332	−183	…	2	…
	E_g_	0.42	half-metal	0.23	0.52	0.54	0.29	0.49

^a^: D_0_ is the metal-nitrogen distance (in Å); D_1_ is the metal-nitrogen (along the Y-axis) distance (in Å); D_2_ is the metal-nitrogen (along the X-axis) distance (in Å); E_ex_ is the exchange energies (*meV*); M_0_ is the magnetic moment per cell (*μ*_*B*_); E_g_ is energy gap for the frameworks.

**Table 2:** Geometric, electronic and magnetic properties of the 1D M_1_M_2_-PPNW and M_1_M_2_-PCNW frameworks.^b^

	TMs	MnCr	MnFe	MnCo	MnCu
PPNW	M_0_	3/4	3/2	3/1	3/1
	E_ex_	−17	6	64	−85
	E_g_	0.28	0.21	0.19	half-metal
PCNW	M_0_	3/4	3/2	3/1	3/1
	E_ex_	−17	11	36	−6
	E_g_	half-metal	metal	0.06	half-metal

^b^: E_ex_ is the exchange energies (*meV*); M_0_ is the magnetic moment for the Mn and Cr/Fe/Co/Cu, respectively, per cell (*μ*_*B*_); E_g_ is energy gap for the frameworks.
